# Neural Correlates of Receiving an Apology and Active Forgiveness: An fMRI Study

**DOI:** 10.1371/journal.pone.0087654

**Published:** 2014-02-05

**Authors:** Sabrina Strang, Verena Utikal, Urs Fischbacher, Bernd Weber, Armin Falk

**Affiliations:** 1 Center for Economics and Neuroscience, University of Bonn, Bonn, Germany; 2 Department of Economics, University of Erlangen-Nürnberg, Nürnberg, Germany; 3 Department of Economics, University of Konstanz, Konstanz, Germany; 4 Thurgau Institute of Economics, Kreuzlingen, Switzerland; 5 Department of Epileptology, University Hospital Bonn, Bonn, Germany; 6 Department of Economics, University of Bonn, Bonn, Germany; University of Milan, Italy

## Abstract

Interpersonal conflicts are a common element of many social relationships. One possible process in rebuilding social relationships is the act of apologizing. Behavioral studies have shown that apologies promote forgiveness. However, the neural bases of receiving an apology and forgiveness are still unknown. Hence, the aim of the present fMRI study was to investigate brain processes involved in receiving an apology and active forgiveness of an ambiguous offense. We asked one group of participants (player A) to make decisions, which were either positive or negative for another group of participants (player B). The intention of player A was ambiguous to player B. In case of a negative impact, participants in the role of player A could send an apology message to participants in the role of player B. Subsequently players B were asked whether they wanted to forgive player A for making a decision with negative consequences. We found that receiving an apology yielded activation in the left inferior frontal gyrus, the left middle temporal gyrus, and left angular gyrus. In line with previous research we found that forgiving judgments activated the right angular gyrus.

## Introduction

Interpersonal relationships are an essential element of our lives in providing support, security and other important social resources. A common challenge to relationships is interpersonal conflict, with the potential of ultimately causing its breakdown. One possible process in rebuilding social relationships is the act of apologizing, since apologies promote forgiveness [1], [2]. Because of its ubiquity, several disciplines have studied the critical role of apologies and their effect on forgiveness.

Behavioral economic research indicates that forgiveness is strongly influenced by apologies [1], [3], [4]. A field experiment by Abeler et al. [5] found that customers of an online store who receive an apology forgive nearly twice as often compared to receiving just a monetary compensation [5]. However, it has also been shown that situational circumstances largely determine whether an apology triggers forgiveness. For example, the intentionality behind the offense turns out to be crucial for forgiveness [1], [6]. Apologies promote forgiving when the underlying intention behind the offense is ambiguous. In case of an obviously intentional offense however, apologies may actually have adverse effects [1].

Research in psychology has mainly used retrospective reports. Participants are asked to recall an experienced transgression and to indicate some transgression related information, such as the impact the transgression had on them or the level of empathy they had towards the offender. These studies found that empathy is a determinant for forgiveness [2], [3], [7]–[10] and suggest that offender focused empathy is a mediator between apologies and forgiveness [11].

Neuroscience research has studied brain activation patterns in the process of forgiving. Brain areas that were found to be associated with forgiveness are the left ventromedial prefrontal cortex, posterior cingulate gyrus and right temporo parietal junction [12]–[15]. However, none of these studies directly measured the impact of forgiving versus not forgiving. Farrow et al. [12] compared empathy with forgiveness judgments and Hayashi et al. [13] compared forgiveness judgements with different perpetrator attitudes and different severities of the transgression. Young and Saxe [14] found a correlation between the right temporo parietal junction and the degree of blame participants ascribed to an offender. They assume that people ascribing more blame are less likely to forgive and vice versa.

No study so far has analysed the neural correlates of receiving apologies. Different approaches were used to explore the neural correlates of forgiveness, which makes it difficult to compare the results. As a consequence, the evidence regarding the neural basis of forgiveness is quite inconclusive. Moreover, and in contrast to the present study, only narrative scenarios were used, which do not involve a consequence for either party and are therefore not ecologically valid.

We aim at improving research on the nature of forgiving by combining behavioral measures and neuroscientific methods. In particular, we study the interplay between apologies and forgiveness combining a behavioral choice paradigm and functional magnetic resonance imaging (fMRI). In a two person game, one person can commit a transgression that has real monetary consequences for another person. The intention of the transgressor is ambiguous to the other person. The transgressor then has the option to apologize. We are interested in the neural processes underlying the effects of receiving an apology and active forgiveness in an ecologically valid setting.

**Figure 1:**
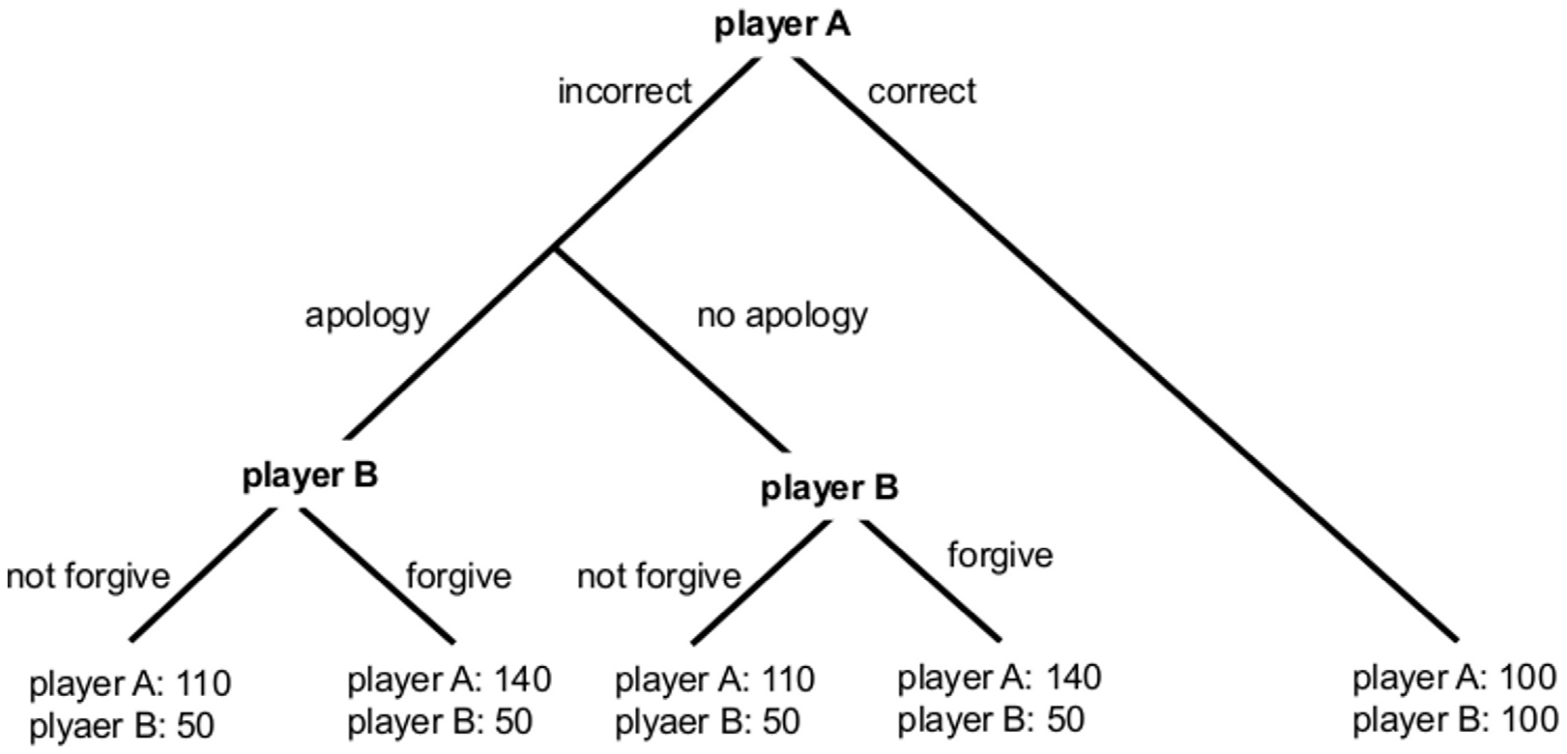
Structure of the game. Player A receives a multiple choice question. In case of a correct answer, players A and B both receive 100 points. In case of a wrong answer, player B only receives 50 points and player A receives more than for giving a correct answer. In this case the exact payoff of player A depends on player B. After answering the question, player A has the option to send a message (which was later categorized in apology or no apology messages) to player B. Player B can decide whether he wants to forgive player A. If he forgives, player A gets 140 points and player B gets 50 points. If he does not forgive, player A receives 110 points and player B receives 50 points.

The design of our experiment is similar to the one used by Fischbacher and Utikal [1]. The game involves two players: Player A (transgressor) and player B (affected person, in the scanner). Player A answers a multiple choice question with four possible answers. Only one of these answers is correct. For the correct answer a fair money allocation is implemented, both players receive 100 points. However, if player A answers the question incorrectly, player A receives more than 100 points and player B receives 50 points only (Figure 1). Further player A has the option to send a message to player B. This message can contain an apology. If player B forgives player A, player A receives 140 points. If player B does not forgive player A, player A receives only 110 points. Therefore, the decision to forgive has a direct impact on player A’s payoff.

Note that we introduced monetary payoffs to induce real consequences for all decisions: transgressions and forgiveness decisions were payoff relevant. Player A’s transgression had consequences for the payoff of both players. Player B’s forgiveness decision influenced player A’s payoff, having no impact on player B’s payoff. It is arguable whether forgiving has some costs in reality. However, by keeping the payoff of player B constant, money-maximizing motives did not confound player B’s forgiveness decision.

The aim of this design was to make the underlying intention behind the offense ambiguous. It was not obvious whether player A answered the question intentionally wrong or whether he did not know the answer. A wrong answer to a very easy question is likely to be intentional. A wrong answer to a very difficult question in contrast might be due to lack of knowledge. Therefore, we chose easy but not trivial questions; herewith we introduced ambiguousness of player A’s intention. The successful implementation of ambiguous intentionality can be inferred from the fact that 46% of the answers in our experiment were correct. When paid for every correct answer, participants solved 87% of the questions correctly (as tested in a pilot experiment). This means that participants were able to solve more answers than they actually did, indicating that they gave some wrong answers intentionally.

Since there are no previous imaging studies about neural correlates of receiving an apology, our hypothesis about brain areas involved in processing apologies is rather explorative. Psychological research suggest an association between apologies, empathy towards the offender and forgiveness [11]. Therefore apologies might increase activation in empathy related brain areas.

The paradigm used by Young and Saxe [14] captures our measurement of forgiveness most closely, therefore we predict to find overlap with their results [14].

## Materials and Methods

### Participants

In the behavioural experiment, 38 (22 women, 22.5±2.89 SD years of age) participants were tested in two sessions. The participants received money depending on the decisions they made during the sessions. Part of the money was paid out immediately after the experiment and the other part four weeks later. The behavioral experiment was conducted in June 2011 at the BonnEconLab (Labor für experimentelle Wirtschaftsforschung) at the University of Bonn using z-Tree [16]. Participants were invited via ORSEE (Online Recruitment System for Economic Experiments) [17]. Thirteen additional participants were invited to categorize all messages written during the behavioral experiment in ‘apology’ or ‘other’ messages.

Thirty-two participants (20 women; 22.5±1.91 SD years of age) took part in the fMRI experiment. They were all native German speakers. Participants had no history of psychiatric or neurological disorders. Written consent was given by all participants according to the Declaration of Helsinki (BMJ 1991; 302: 1194) and the study was approved by the ethics committee of the University of Bonn. Participants from the same subject pool (BonnEconLab) as used for the behavioral experiment were invited to the fMRI experiment. All participants received a ten Euro show-up fee at the end of the experiment. Participants were informed that in addition they and the players A would receive their payoff depending on one randomly chosen decision they made during the experiment. The additional payoff was paid some weeks later. The experiment was conducted in June and July of 2011 at the Life and Brain Center Bonn, Department of NeuroCognition and Clinic of Epileptology, Bonn, Germany.

### Experimental Design

#### Behavioral experiment

Participants had to accomplish two consecutive phases in the experiments. Phase 1 was a training. During five periods, half of the participants played the role of player A and the other half were player B. Player A received a multiple choice question. If he gave the correct answer, he received 100 points and player B received 100 points as well. If he answered the question incorrectly, player B received 50 points and player A’s payoff depended on the decision of player B. Before player B’s decision, player A had the option to send a message to player B. Player B was presented with the question player A received and learned about the result of player A. In case of a correct answer, player B received 100 points and the game ended. In case of a wrong answer, the following procedure applied: Player B further received the message from player A. Player B could decide whether to forgive player A. If player B forgave player A giving a wrong answer, player A received 140 points. In case player B did not forgive player A, player A received 110 points (Figure 1).

Prior to the experiment, participants received an instruction manual including four comprehension questions to ensure that all participants had understood the task.

We used a perfect stranger matching to allocate participants for each period. This means that in each period, player A played against a different player B. At the end of the first experiment one of the participants threw a dice to determine which of the five periods was paid. The exchange rate of this experiment was 1 points = 5 cent. Participants received the money earned during this experiment at the end of the session. Phase 1 was a training; participants had the chance to get used to the task and additionally to learn what kind of messages might be useful to send.

In phase 2, the task was almost the same as in phase 1, but now all participants were players A and ten periods were played. Participants were further informed about the following modified rules: players B will take part in the experiment at a later date, players B will not receive the entire messages sent by players A but a categorized version, and not all periods played by player A are transferred to players B but only a sample selected by the experimenters. In addition, participants were told that after the decisions of players B, a payoff-relevant period will be randomly selected, the exchange rate is 1 point = 30 cent, and that the payoff is about four weeks after the session. A set of the answers given in this experiment was presented to the participants of the fMRI experiment.

In a pilot experiment, participants had to answer ten multiple choice questions of the same question pool as in both previous experiments. However, this time, participants received 2 points for each question they answered correctly. The exchange rate was 1 point = 5 cent. Participants received their payoff at the end of the session. We implemented this pilot experiment to study how many questions the participants were actually able to solve on average.

#### fMRI experiment

**Figure 2:**
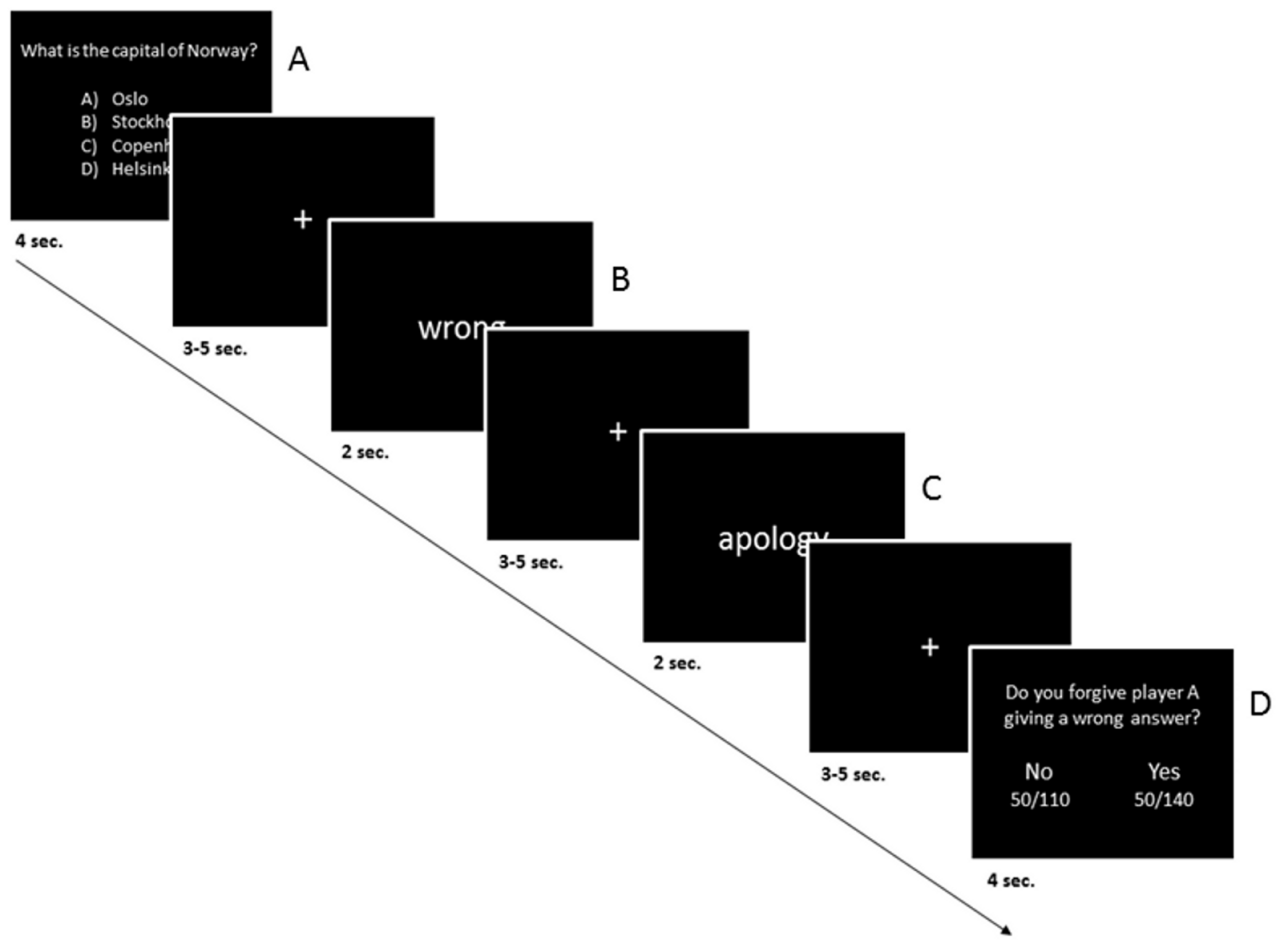
Experimental design. Time course of stimulus presentation. Inter-stimulus-interval = jittered between 3 to 5 seconds, A: 120 multiple choice questions answered by players A were presented to player B. B: 30 of the questions were answered right and 90 were answered wrong. In case of a right answer, the trial ended. Otherwise C and D followed. C: of the 90 incorrectly answered questions, half were followed by a message categorized as an apology. D: player B could either forgive or not forgive player A.

All participants received detailed information about fMRI in general, exclusion criteria, the experiment, and the data handling. In addition, all subjects received detailed written and verbal instructions, and completed the same comprehension questions as in the behavioral experiment. In order to get an impression of what kind of messages were categorized as an apology players B participating in the fMRI experiment received six randomly chosen example messages of the category ‘apology’ before the experiment started. Furthermore, participants were shown all ‘apology’ messages after the experiment. The participants of the fMRI experiment played the role of player B. They received a set of 120 multiple choice questions answered by players A. The question set contained 30 correct answered questions and 90 incorrect answered questions. Of the 90 incorrect answered questions, 45 were followed by a message categorized as an apology. The multiple choice questions were presented for four seconds. After a time interval of four seconds (jittered between three and five seconds), Player B was informed of whether the answer of player A was right or wrong. This information was displayed for two seconds. After a time interval of four seconds (jittered between three and five seconds), player B received either an apology or no apology information. In case player A sent an apology, player B received an ‘apology message’. In case player A did not send a message at all, or a message which was not categorized as an apology, player B received a no apology message. The apology and no apology information were presented for two seconds. Four seconds (jittered between three and five seconds) later, player B had to decide whether he wanted to forgive player A for giving a wrong answer. The next trial started after a response of player B. The response time window was four seconds and the inter trial interval was four seconds, jittered between three and five seconds (Figure 2).

### Imaging Protocol

Scanning was performed on a 3 Tesla Trio Scanner (Siemens, Erlangen, Germany) using an 8-channel head coil. Functional data was acquired using EPI-sequences with a repetition time (TR) of 2.5 s, an echo time (TE) of 30 ms, and a flip angle of 90 degrees. Each volume comprised 37 slices acquired in an axial orientation covering all of the brain, including the midbrain, but sparing parts of the cerebellum. The presentation of the task and recording of behavioral responses were performed with Presentation® software version 14.9 (Neurobehavioral Systems, Albana, Canada). Subjects saw the experiment via video goggles (Nordic NeuroLab, Bergen, Norway) and gave their responses by response grips (Nordic NeuroLab, Bergen, Norway) using the index fingers of both hands.

### fMRI Analyses

fMRI data of 29 participants were analyzed using SPM8 (Wellcome Department of Imaging Neuroscience, London, UK). Data sets of two participants were excluded due to excessive head motion and one because of technical problems. The following pre-processing steps were applied: slice time correction, motion correction, linear trend removal, high pass temporal filtering with a filter size of 128 seconds, spatial smoothing using a Gaussian kernel with full-width at half-maximum (FWHM) of 8 mm and spatial normalization by corregistering the functional data with the individual structural data and then transforming the data into the Montreal Neurological Institute (MNI) template space.

For each participant, brain activation was estimated using a general linear model. Seven onset regressors were defined to estimate activation caused by the task. These onset regressors were: 1) question presentation, 2) correct answer, 3) wrong answer, 4) apology, 5) no apology, 6) forgiveness and 7) no forgiveness. In addition, six motion regressors were defined: three translation regressors, x, y, and z, and three rotation regressors, pitch, roll, and yaw. The regressors were convolved with a hemodynamic response function (HRF) in order to consider for the hemodynamic response of the measured blood oxygenation level dependent (BOLD) signal. For each onset regressor parameter estimates were generated and the contrasts ‘apology versus no apology’ and ‘forgiveness versus no forgiveness’ were calculated in the first level analyses. The obtained contrast images were transferred to the second level random effects analyses. In the second level analyses, one sample t-tests for the apology and forgiveness contrasts were conducted.

## Results

### Behavioral Results

In phase 2 players A wrongly answered on average 54% of the multiple choice questions. 74% of these wrongly answered questions were followed by a message. 31% of the messages were categorized as ‘apology’ and 43% were categorized as ‘no apology’. An overview of the behavioral results of the experiment is given in Table 1.

**Table 1:** Overview of the answers of player A (*N* = 38).

Questions solved	Questions not solved			Total
176 (46%)	204 (54%)			380
	No message	Message		
		No apology	Apology	
	53 (26%)	88 (43%)	63 (31%)	

Number of solved and unsolved questions, number of written messages and number of categorized apologies, as categorized by an independent subject group. The numbers in brackets represent the percentages.

**Figure 3:**
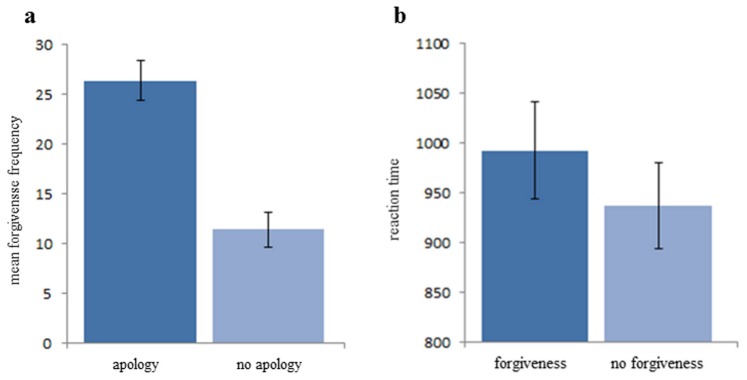
Forgiveness frequency and reaction times in the fMRI experiment. Error bars indicate SEM a, mean forgiveness frequency after apology and no apology messages. Participants forgave significantly more after an apology than after no apology. b, mean reaction time for forgiveness decision and no forgiveness decisions. Reaction times were significantly longer for forgiveness than for no forgiveness.

Players B forgave more often after an apology message (mean: 26.38, ±1.99 SEM) compared to a no apology message (mean: 11.45, ±1.74 SEM; z: 6.35; p<0.01; Figure 3a). The reaction times were longer for forgiving (mean: 991.99 ms, ±48.74 SEM) in comparison to not forgiving (mean: 937.10 ms, ±43.28 SEM). This difference was significant (T: 2.743; p<0.05; Figure 3b).

### fMRI Results

**Figure 4:**
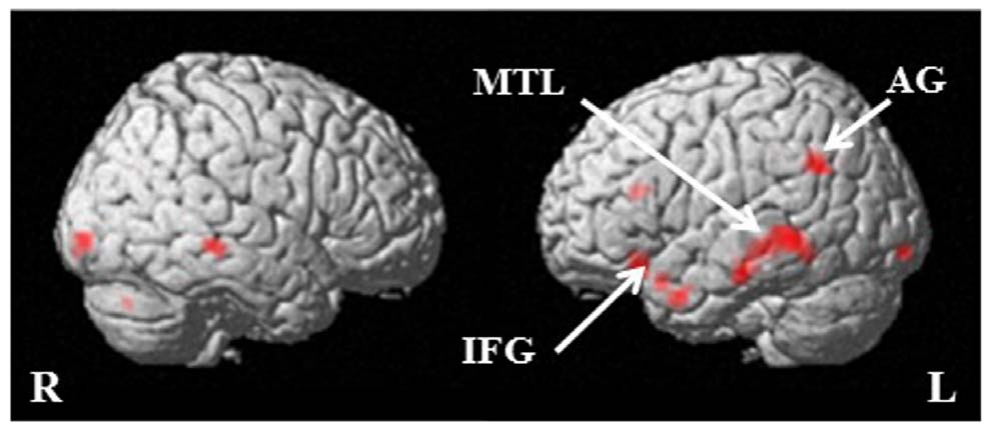
‘Apology’ versus ‘no apology’ contrast. Displayed at *p*<0.001 uncorrected, on the single subject structural SPM template. The red color represents foci that show activation for the contrast ‘apology’ versus ‘no apology’. The following abbreviations are used: IFG (inferior frontal gyrus), MTG (middle temporal gyrus), AG (angular gyrus), R (right hemisphere) and L (left hemisphere).

At the whole brain level *p*<0.001, the contrast ‘apology’ versus ‘no apology’ revealed activation in the left middle temporal gyrus (k = 152; peak voxel at −63, −46, −5; t = 5.03; *p*_FWE_ <0.05; Figure 4; see Table S1 for all whole brain level results at *p*<0.001 uncorrected). Since the ‘apology’ versus ‘no apology’ contrast is rather explorative we further used a more liberal threshold of *p*<0.005 with a 10 voxels threshold extent [18] (Table 2). At this threshold increased activation in the left middle temporal gyrus, the left angular gyrus and the inferior frontal gyrus are observed. Activation in these regions is also small volume corrected significant (Table S2). Areas with higher activation for ‘no apology’ than for ‘apology’ were not observed.

**Table 2:** ‘Apology’ versus ‘no apology’ contrast.

Region	Laterality	MNI coordinates			Cluster size *k_E_*	*t*	*p*-value
		x	y	z			
Middle temporal gyrus	L	−63	−46	−5	313	5.03	*p*<0.01
Angular gyrus	L	−51	−55	31	127	4.55	*p*<0.05
Inferior frontal gyrus (orbital part)	L	−27	14	−23	167	4.33	*p*<0.05

Whole brain activation for the contrast no ‘apology’ versus ‘no apology’ (with *p*_uncorrected_<0.005, 10 voxels threshold extent, whole brain). Brain regions are labeled according to the automated anatomic labeling toolbox for SPM8.

**Figure 5:**
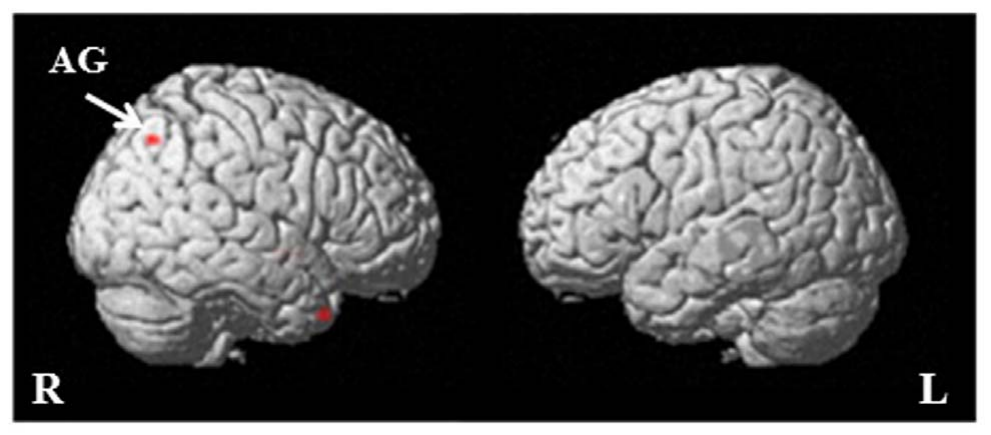
‘Forgiveness’ versus ‘no forgiveness’ contrast. Displayed at *p*<0.001 uncorrected, on the single subject structural SPM template. The red color represents the focus that showed activation for the contrast ‘forgiveness’ versus ‘no forgiveness’. The following abbreviations are used: AG (angular gyrus), L (left hemisphere) and R (right hemisphere).

For the contrast ‘forgiveness’ versus ‘no forgiveness’ we created an anatomical mask based on the results of Young and Saxe [14]. They reported the right temporo parietal junction (TPJ) to be involved in forgiveness judgments. Since the TPJ consists of several sub regions we determined the sub region they found to be activated; the right angular gyrus. Hence, we used the right angular gyrus for the small volume analysis of the forgiveness contrast. The ‘forgiveness’ versus ‘no forgiveness’ contrast revealed a small volume corrected significant difference (k = 7, peak voxel at 39, −67, 46; t = 3.58, *p*_FWE_ <0.05; Figure 5; see Table S3 for whole brain level results at *p*<0.001 uncorrected). The contrast ‘no forgiveness’ versus ‘forgiveness’ revealed no significant activation.

## Discussion

This study is a first attempt to investigate the neural correlates of receiving an apology and active forgiveness in an ecologically valid setting. We introduced a new paradigm combining fMRI with incentivized behavioral measures. The design of our study allowed us not only to compare changes in neural activity associated with forgiveness versus no forgiveness, but also activity associated with receiving an apology within the same setting. Our data show that receiving an apology leads to increased activity in a left lateralized network containing frontal, temporal and parietal regions. Forgiveness revealed increased activation in the right angular gyrus. In line with previous research, participants were more willing to forgive after an apology [1].

Activation in a network of frontal, temporal and parietal regions is often found in empathy processes [19], [20]. Empathy includes emotional as well as cognitive processes [21]. By simulating the emotional experience of others we can intuitively understand what the other person feels. The human mirror neuron system, encompassing the inferior frontal gyrus (IFG), temporal lobes and inferior parietal cortex (IPC), was shown to be involved in this simulation process [19], [22]. Schulte-Rüther et al. [20] emphasized a specific role of the IFG in this emotional process of empathy. However, we can also share feelings on a more cognitive level through mentalizing. Medial prefrontal cortex (MPFC), temporal poles, superior temporal sulcus (STS) and the temporo-parietal junction (TPJ) are thought to play a role in mentalizing processes [23], [24].

In our study most of the empathy related brain regions showed increased activation when receiving an apology, namely the left IFG, left MTG and left angular gyrus (subpart of the TPJ). However, we did not find any activation in the MPFC, STS and IPC. Thus, the results provide partial evidence for a link between receiving an apology and empathy. Psychological literature suggests a link between apologies, empathy and forgiveness [11]. According to McCullough et al. [11], the sequence of apology-empathy-forgiving is an important mechanism that helps to maintain continuity in close relationships that have been damaged by an offense.

If empathy processes are involved when receiving an apology one open question is why we did not find any activation in the MPFC, STS and IPC. Especially the MPFC is frequently reported to be associated with empathy, activity in this region was shown to correlate with self-reported empathy and trait empathy [25]. There are at least three possible explanations.

First, empathy has no generally accepted definition; its different phenomena remain controversial [26]–[28]. In the social cognitive neuroscience literature empathy is mostly defined as the ability to share the feelings of others [29]. According to some psychologists empathy also includes feelings of warmth, compassion, concern for others and sympathetic responding [28], [30]. In our study participants had no information about the feelings of the other person; therefore it is difficult to share these feelings. However receiving an apology might trigger concern for the other person or sympathetic responses. Thus in our study the responses to apologies rather fit the psychological concept of empathy. The deviation in empathy concepts might explain why we did not find activation in all areas associated with the social cognitive neuroscience definition of empathy. More neuroimaging research on empathic concern and sympathetic response is needed in order to learn whether the corresponding neural correlates are different from the established empathy areas.

Second, in contrast to most fMRI empathy studies we did not use any pictures in our paradigm. The STS is frequently reported to respond to facial stimuli [31], [32]. According to the ‘perception-action model of empathy’ early visual information of an action is processed in the STS [19], [26]. Thus, the difference in stimuli might explain the lack of STS activity in our study. However, this does not explain the absence of MPFC and IPC activation.

Third, we have no data about the phenomenological experience of our participants. Thus, our data does not conclusively show that participants actually experienced empathy. Other cognitive processes in response to receiving an apology cannot be fully excluded. By asking participants about their experience when receiving an apology it might be possible to investigate whether the phenomenological experience matches the neuronal data.

In summary, our results point at a possible involvement of empathy processes when receiving an apology. However, since this is the first neuroimaging study investigating the neural correlates of receiving an apology more research is needed to confirm this result.

Our results indicate a recruitment of the right angular gyrus in forgiving. The right angular gyrus is a sub region of the temporo-parietal junction (TPJ). TPJ activation has been found with several paradigms that measure attention [33], memory [34] or social cognition [24]. Recent studies suggest that the right angular gyrus has a distinct role in social cognition [35], [36]. In a meta-analysis activation in the right angular gyrus was found to be associated with complex social functions [36]. Enhancing TPJ activation by tDCS stimulation leads to improved social cognition [37], decreasing activation by rTMS reduces parochial punishment [38]. Since forgiveness is a highly social process, our results provide further support for a distinct role of the right angular gyrus in social cognition.

The right angular gyrus was also found to be involved in forgivability judgments by Young & Saxe [14]. In their study participants with high activity in this region assigned less blame than subjects with low activity to the same harmful outcomes. Thus, high activation was associated with less blame which is assumed to result in an increased likelihood to forgive.

Other imaging studies investigating forgiveness found the posterior cingulate gyrus and the left ventromedial prefrontal cortex to be activated during forgiveness [12], [13]. However, they used different experimental settings which might explain this discrepancy.

Farrow et al. [12] compared forgiveness judgments with empathic and social reasoning judgments. Thus, the content of the judgments made in the three conditions in their experiment differed. They did not discern between forgiving and not forgiving solely. Their results rather indicate an involvement of the posterior cingulate gyrus in making judgments about transgression in comparison to empathic or social reasoning judgments, but do not indicate a direct involvement in the decision to forgive. Hayashi et al. [13] asked their subjects to judge the forgivability of one scenario under four different conditions. They varied two factors; the seriousness of the transgression and the honesty of the transgressors. The left ventromedial prefrontal cortex showed an interaction between the two factors. A correlational analysis between the forgiveness scores and the regional cerebral blood flow was conducted in order to identify brain regions involved in the decision to forgive but yielded no significant results.

Our paradigm allowed us to investigate active forgiveness under conditions in which the intention of the offender is ambiguous and in which forgiving has an actual impact on the incentive of the offender. The results indicate that receiving an apology leads to increased activity in a left lateralized network containing frontal, temporal and parietal regions. During forgiving the right angular gyrus was activated.

## Supporting Information

Table S1 **‘Apology’ versus ‘no apology’ contrast.** Whole brain activation for the contrast no ‘apology’ versus ‘no apology’ (*p*_uncorrected_<0.001, whole brain).
(DOCX)

Table S2 **‘Apology’ versus ‘no apology’ contrast small volume analysis.** A priori-created anatomical masks of established empathy regions for a region of interest analysis [16]–[20]. Small volume corrected activation for the contrast no ‘apology’ versus ‘no apology’ (with *p*_uncorrected_<0.001, whole brain).
(DOCX)

Table S3 **‘Forgiveness’ versus ‘no forgiveness’ contrast.** Whole brain activation for the contrast no ‘apology’ versus ‘no apology’ (with *p*_uncorrected_<0.001, whole brain).
(DOCX)
