# Supplementary material for: Neural Correlates of Receiving an Apology and Active Forgiveness: An fMRI Study
Source: PLoS One. 2014 Feb 5;9(2):e87654. doi: 10.1371/journal.pone.0087654 (PMC3914861; doi:10.1371/journal.pone.0087654)
Supplement: Table S1 — ‘Apology’ versus ‘no apology’ contrast. Whole brain activation for the contrast no ‘apology’ versus ‘no apology’ (p uncorrected<0.001, whole brain). (DOCX) [file pone.0087654.s001.docx]

**Table S1:** ‘Apology’ versus ‘no apology’ contrast. Whole brain activation for the contrast no ‘apology’ versus ‘no apology’ (*p*_uncorrected_ < 0.001, whole brain).

| **Region** | **Laterality** | **MNI coordinates** | | | **Cluster size *k_E_*** | ***t*** |
| --- | --- | --- | --- | --- | --- | --- |
|  |  | **x** | **y** | **z** |  |  |
| Middle temporal gyrus | L | −63 | −46 | −5 | 152 | 5.03 |
|  |  | −57 | 8 | −29 | 16 | 4.34 |
| Inferior occipital gyrus | R | 33 | −94 | −5 | 31 | 4.58 |
| Cerebellum | L | −51 | −55 | −31 | 42 | 4.55 |
| Inferior frontal gyrus (orbital part) | L | −27 | 14 | −23 | 29 | 4.33 |
|  |  | −45 | 26 | −17 | 28 | 4.21 |
| Middle temporal gyrus | R | 66 | −34 | −8 | 20 | 4.22 |
| Inferior occipital gyrus | L | −24 | −97 | −8 | 12 | 4.00 |
| Middle cingular gyurs | L | −3 | −37 | 34 | 8 | 3.93 |
| Inferior frontal gyrus (triangular part) | L | −51 | 23 | 19 | 13 | 3.86 |
| Cerebellum | R | 42 | −76 | −32 | 5 | 3.74 |
| Precuneus | L | −3 | −67 | 34 | 7 | 3.66 |

Only clusters with >5 voxels are reported. Brain regions are labeled according to the automated anatomic labeling toolbox for SPM8.
