# Supplementary material for: Neural Correlates of Receiving an Apology and Active Forgiveness: An fMRI Study
Source: PLoS One. 2014 Feb 5;9(2):e87654. doi: 10.1371/journal.pone.0087654 (PMC3914861; doi:10.1371/journal.pone.0087654)
Supplement: Table S2 — ‘Apology’ versus ‘no apology’ contrast small volume analysis. A priori-created anatomical masks of established empathy regions for a region of interest analysis [16]–[20]. Small volume corrected activation for the contrast no ‘apology’ versus ‘no apology’ (with p uncorrected<0.001, whole brain). (DOCX) [file pone.0087654.s002.docx]

**Table S2:** ‘Apology’ versus ‘no apology’ contrast small volume analysis. A priori-created anatomical masks of established empathy regions for a region of interest analysis [16–20]. Small volume corrected activation for the contrast no ‘apology’ versus ‘no apology’ (with *p*_uncorrected_ < 0.001, whole brain).

| **Region** | **Laterality** | **MNI coordinates** | | | **Cluster size *k_E_*** | ***t*** | ***p*-value** (FEW-corrected) |
| --- | --- | --- | --- | --- | --- | --- | --- |
|  |  | **x** | **Y** | **z** |  |  |  |
| Inferior frontal gyrus (triangular part) | L | −51 | 23 | 19 | 13 | 3.86 | *p* < 0.05 |
| Inferior frontal gyrus (orbital part) | L | −27 | 15 | −23 | 6 | 4.33 | *p* < 0.05 |
|  | L | −45 | 26 | −17 | 21 | 4.21 | *p* < 0.05 |
| Middle temporal gyrus | L | −63 | −46 | −5 | 138 | 5.03 | *p* < 0.01 |
| Temporal pole (middle part) | L | −54 | 8 | −32 | 5 | 4.31 | *p* < 0.05 |
| Temporal pole (superior part) | L | −45 | 26 | −20 | 6 | 4.10 | *p* < 0.05 |
|  | L | −39 | 17 | −26 | 20 | 4.04 | *p* < 0.05 |
| Angular gyrus | L | −51 | −55 | 31 | 35 | 4.55 | *p* < 0.01 |

Brain regions are labeled according to the automated anatomic labeling toolbox for SPM8.
