# Supplementary material for: Neural Correlates of Receiving an Apology and Active Forgiveness: An fMRI Study
Source: PLoS One. 2014 Feb 5;9(2):e87654. doi: 10.1371/journal.pone.0087654 (PMC3914861; doi:10.1371/journal.pone.0087654)
Supplement: Table S3 — ‘Forgiveness’ versus ‘no forgiveness’ contrast. Whole brain activation for the contrast no ‘apology’ versus ‘no apology’ (with p uncorrected<0.001, whole brain). (DOCX) [file pone.0087654.s003.docx]

**Table S3:** ‘Forgiveness’ versus ‘no forgiveness’ contrast. Whole brain activation for the contrast no ‘apology’ versus ‘no apology’ (with *p*_uncorrected_ < 0.001, whole brain).

| **Region** | **Laterality** | **MNI coordinates** | | | **Cluster size *k_E_*** | ***t*** |
| --- | --- | --- | --- | --- | --- | --- |
|  |  | **x** | **y** | **z** |  |  |
| Putamen | R | 18 | 2 | −8 | 6 | 4.70 |
| Temporal pole | R | 39 | 17 | −38 | 5 | 4.08 |
| Angular gyrus | R | 39 | −67 | 46 | 7 | 43.58 |

Only clusters with >5 voxels are reported. Brain regions are labeled according to the automated anatomic labeling toolbox for SPM8.
